# Efficacy and Safety of Re-administration of Epidermal Growth Factor Receptor-Tyrosine Kinase Inhibitor (EGFR-TKI) After EGFR-TKI-Induced Interstitial Lung Disease (CS-Lung-005)

**DOI:** 10.1007/s00408-023-00669-9

**Published:** 2024-01-24

**Authors:** Nobuhiro Kanaji, Eiki Ichihara, Takaaki Tanaka, Takashi Ninomiya, Toshiyuki Kozuki, Nobuhisa Ishikawa, Kazuya Nishii, Hiroyasu Shoda, Kakuhiro Yamaguchi, Keita Kawakado, Yuko Toyoda, Masaaki Inoue, Nobuyuki Miyatake, Naoki Watanabe, Takuya Inoue, Hitoshi Mizoguchi, Yuta Komori, Kazuki Kojima, Norimitsu Kadowaki

**Affiliations:** 1 Department of Internal Medicine, Division of Hematology, Rheumatology and Respiratory Medicine, Faculty of Medicine, Kagawa University, 1750-1 Ikenobe, Miki-cho, Kita-gun, Kagawa, 761-0793 Japan; 2 Department of Allergy and Respiratory Medicine, Okayama University Hospital, Okayama, Japan; 3 Department of Thoracic Oncology and Medicine, National Hospital Organization Shikoku Cancer Center, Ehime, Japan; 4 Department of Respiratory Medicine, Hiroshima Prefectural Hospital, Hiroshima, Japan; 5 Department of Respiratory Medicine, National Hospital Organization Iwakuni Clinical Center, Yamaguchi, Japan; 6 Department of Respiratory Medicine, Hiroshima Citizens Hospital, Hiroshima, Japan; 7 Department of Respiratory Medicine, Hiroshima University Hospital, Hiroshima, Japan; 8 Department of Internal Medicine, Division of Medical Oncology and Respiratory Medicine, Shimane University Faculty of Medicine, Shimane, Japan; 9 Department of Internal Medicine, Japanese Red Cross Kochi Hospital, Kochi, Japan; 10 Department of Chest Surgery, Shimonoseki City Hospital, Shimonoseki, Japan; 11 Department of Hygiene, Faculty of Medicine, Kagawa University, Kagawa, Japan

**Keywords:** Epidermal growth factor inhibitor, Interstitial lung disease, Pneumonitis, Rechallenge, Response rate, Survival

## Abstract

**Purpose:**

This study investigated the safety and efficacy of epidermal growth factor receptor (EGFR)-tyrosine kinase inhibitor (TKI) re-administration after recovery from EGFR-TKI-induced interstitial lung disease (ILD).

**Methods:**

This multicenter retrospective study collected data from consecutive advanced NSCLC patients who underwent EGFR-TKI re-administration after recovery from EGFR-TKI-induced ILD.

**Results:**

Fifty-eight patients were registered. The grades of initial TKI-induced ILD were grade 1 to 4. TKIs used for re-administration were erlotinib for 15 patients, osimertinib for 15, gefitinib for 14, afatinib for 13 patients, and dacomitinib for 1 patient. ILD recurred in 13 patients (22.4%), comprising 3 patients with grade 1, 6 patients with grade 2, and 4 patients with grade 3. No significant associations were found between ILD recurrence and age, smoking history, performance status, time from initial ILD to TKI re-administration, or concomitant corticosteroid use. However, the incidence of ILD recurrence was high in cases of repeated use of gefitinib or erlotinib or first time use of osimertinib at TKI re-administration. The ILD recurrence rate was lowest in patients treated with first time use of gefitinib (8%) or erlotinib (8%), followed by patients treated with repeated use of osimertinib (9%). The response rate, median progression-free survival by TKI re-administration, and median overall survival were 55%, 9.6 and 84.8 months, respectively.

**Conclusion:**

This study showed that EGFR-TKI re-administration is a feasible and effective treatment for patients who recovered from EGFR-TKI-induced ILD. Our results indicate that re-administration of EGFR-TKI is an important option for long-term prognosis after recovery from EGFR-TKI-induced ILD.

## Introduction

Epidermal growth factor receptor (EGFR)-tyrosine kinase inhibitors (TKIs) are currently the most effective treatment for advanced non-small cell lung cancer (NSCLC) with *EGFR* mutations and can result in marked tumor shrinkage. However, EGFR-TKI-induced interstitial lung disease (ILD) can occur as a critical, potentially lethal, adverse event. Because EGFR-TKI-induced ILD can be severe, once it occurs, EGFR-TKIs are generally discontinued and corticosteroids are used as needed.

Several clinical trials have reported a frequency of EGFR-TKI-induced ILD ranging from 1.3 to 5.3% [1–3]. A higher frequency of ILD was reported in osimertinib-treated patients in the FLAURA trial (4% in osimertinib vs 2.2% in gefitinib or erlotinib groups) and in Asian subset (6% in osimertinib vs 2% in gefitinib or erlotinib groups) [4, 5]. In the Japanese subset in FLAURA, the frequency of osimertinib-induced ILD was even higher, with 8 (12.3%) patients in the osimertinib group and one (1.8%) in the gefitinib group [6]. Even in patients treated with first- and second-generation EGFR-TKIs, a high incidence of ILD (17/196 patients, 8.6%) was reported in real-world evidence in Japan [7].

In a retrospective study of 11 patients who received EGFR-TKI after recovery from EGFR-TKI-induced ILD, ILD recurred in one patient [8]. One study of five cases treated with EGFR-TKI re-administration reported grade 3 ILD in one case [7]. Another study of four cases reported no ILD recurrence [9]. In these case series studies, the overall response rates to EGFR-TKI re-administration were very high (75–100%) [7–9]. While cases successfully treated with EGFR-TKI re-administration have been reported, the number of cases reported so far have been small and thus the frequency and severity of recurrent EGFR-TKI-induced ILD are not well understood. There are currently no guidelines on whether EGFR-TKIs should be used again during the course of treatment after EGFR-TKI-induced ILD. Unmet needs exist in this population.

We conducted this study to obtain useful information regarding the benefits and drawbacks of EGFR-TKI re-administration by evaluating the efficacy and adverse events of EGFR-TKI re-administration in patients after recovery from EGFR-TKI-induced ILD.

## Materials and Methods

### Patients and Study Design

In this multicenter, retrospective CS-Lung-005 study, patients who met the following criteria were included: (1) patients with pathologically confirmed recurrent or advanced NSCLC with sensitizing EGFR mutation at diagnosis, (2) patients who experienced ILD caused by EGFR-TKI, (3) patients who discontinued EGFR-TKI because of EGFR-TKI-induced ILD, (4) patients who recovered from EGFR-TKI-induced ILD, and (5) patients who received EGFR-TKI again from April 1, 2007 to July 31, 2022. The type of EGFR-TKI, therapeutic line of EGFR-TKI, and concomitant medications were not specified for either first or second administration of EGFR-TKI. Patients in which initial TKI was continued after the onset of ILD were excluded in this study.

The primary endpoint was the frequency of recurrent ILD after re-administration of EGFR-TKI. The secondary endpoints were grade of ILD, adverse events other than ILD, response rate, progression-free survival (PFS), and overall survival (OS).

This study was approved by the Research Ethics Committee of Kagawa University Faculty of Medicine (no. 2022-117) and Japanese Red Cross Kochi Hospital (no. 432). We obtained informed consent for the use of patient information through an opt-out form or a written form because of the retrospective nature of the study.

### Evaluation of EGFR-TKI-Induced ILD

The evaluation of ILDs on high-resolution CT was performed in accordance with the American Thoracic Society/European Respiratory Society/Japanese Respiratory Society/Latin American Thoracic Society statement [10, 11]. The commonly described patterns of drug-related pneumonitis include nonspecific interstitial pneumonia, organizing pneumonia, diffuse alveolar damage (DAD), hypersensitivity pneumonitis, and simple pulmonary eosinophilia [12]. EGFR-TKI-induced ILDs were diagnosed by respiratory specialists at each institute on the basis of the patients’ clinical features, radiologic findings, and bronchoscopic examination when available. The toxicity severity of pneumonitis was determined using the Common Terminology Criteria for Adverse Events version 5.

### Statistical Analysis

PFS was defined as the time between the start of re-administration of EGFR-TKI and the diagnosis of disease progression or death. OS was defined as the time between the date of diagnosis or recurrence and date of death from any cause. PFS and OS curves were constructed by the Kaplan–Meier method, and differences in survival were compared using the log-rank test. Fisher’s exact test, chi-square test, and Student’s *t* test were used to analyze patient characteristics. All statistical analyses were conducted using BellCurve for Excel v.4.05 (Social Survey Research Information Co., Ltd., Japan).

## Results

### Patient Characteristics and Status of Initial TKI-Induced ILD

Fifty-eight patients were registered. The characteristics of the patients at diagnosis of lung cancer and initial EGFR-TKI treatment that induced ILD are shown in Table 1. Of the 36 patients who were treated with osimertinib, 24 received osimertinib as first-line therapy. Information on initial TKI-induced ILD is summarized in Table 2. The median onset of initial TKI-induced ILD was 61 days. Most cases were grade 1 or 2, while 11 cases were grade 3, and 1 case was grade 4. Thirty patients were treated with corticosteroids.

**Table 1:** Patient and tumor characteristics

Characteristic	Total (*n* = 58)
Age at diagnosis for lung cancer, average (range)	71 (39–85)
Gender, male/female	25/33
Smoking history, smoker/never smoker	29/29
Pack-years in smokers, average (range)	27 (0.5–144)
Histology, adenocarcinoma/NSCLC	55/3
Type of EGFR mutation, exon19 del/L858R/exon19del + T790M/unknown	27/28/1/2
Clinical stage, III/IV/relapse	6/35/17
Initial EGFR-TKI which induced ILD	
Gefitinib	6
Erlotinib*	8
Afatinib*	8
Osimertinib	36
Therapeutic line, 1/2/3/4/5/6th	36/13/2/4/1/2
Response, PR/SD/PD/NE	37/9/1/11

*ILD* interstitial lung disease, *NE* not evaluated, *PD* progressive disease, *PR* partial response, *SD* stable disease

*One patient was treated with addition of bevacizumab

**Table 2:** Initial TKI-induced ILD and TKI re-administration-induced ILD

Characteristic	Initial TKI-induced ILD (*n* = 58)	TKI re-administration-induced ILD (*n* = 13)
Incidence	NA	13/58 (22.4%)
Onset, days from TKI, median	61 (4–537)	66 (3–446)
CTCAE Grade, 1/2/3/4	26/20/11/1	3/6/4/0
Radiological pattern of TKI-induced ILD		
DAD	4	0
Non-DAD	53	13
HP/OP/AEP/NSIP/unclassifiable	11/23/8/4/7	3/5/1/2/2
Undetermined	1	0
Treatment with corticosteroid, yes (with pulse therapy)/no	30 (8)/28	9 (4)/4

*AEP* acute eosinophilic pneumonia, *DAD* diffuse alveolar damage, *HP* hypersensitivity pneumonia, *ILD* interstitial lung disease, *NA* not assessed, *NSIP* non-specific interstitial pneumonia, *OP* organizing pneumonia, *TKI* tyrosine kinase inhibitor

### Status of TKI Re-administration and ILD Recurrence Rate

EGFR-TKIs used at re-administration varied and included gefitinib for 14 cases, erlotinib for 15, afatinib for 13, and osimertinib for 15 (Table 3). Concomitant corticosteroids were administered in 17 patients, and concomitant anti-angiogenic agents were administered in 3 patients. ILD was induced in 13 patients (22.4%) after TKI re-administration (Table 2). 95% interval of incidence of ILD was calculated as 11.68–33.15%. The grade of TKI re-administration-induced ILD was grade 1 in 3 patients, grade 2 in 6 patients, and grade 3 in 4 patients. There were no patients with grade 4 or 5 ILD. In all cases, the radiographic pattern was non-DAD. Corticosteroids were used for nine patients. Among 4 patients with grade 3 ILD, three improved and one died of cancer progression during steroid therapy. There were no other serious adverse events by re-administration of TKI.

**Table 3:** Characteristics at EGFR-TKI re-administration

Characteristic	Total (*n* = 58)	TKI re-administration-induced ILD ( +) (*n* = 13)	TKI re-administration-induced ILD ( −) (*n* = 45)	*P* value
Age*, average (range)	71 (39–85)	69 (57–84)	71 (39–85)	0.96
Gender, male/female	25/33	9/4	16/29	0.05
Smoking history, smoker/never smoker	29/29	8/5	21/24	0.53
Pack-years in smokers, average (range)	27 (1–144)	33.5 (0.5–54)	27 (1.25–144)	0.53
ECOG performance status, 0/1/2	12/39/7	1/10/2	11/29/5	0.42
Type of EGFR mutation, exon19del/L858R/exon19del + T790M/unknown	27/28/1/2	5/7/0/1	22/21/1/1	0.75
EGFR T790M, yes/no	6/52	3/10	3/42	0.12
Clinical stage, III/IV/relapse	6/35/17	1/5/7	5/30/10	0.09
Status of TKI re-administration				
TKI used at re-administration				
Gefitinib	14	4	10	0.88
Erlotinib^a,b^	15	3^#^	12^$^	
Afatinib	13	2	11	
Dacomitinib	1	0	1	
Osimertinib	15	4	11	
Same/Different from initial TKI	17/41	6/7	11/34	0.17
Individual TKI administration pattern				
Gefitinib as first time use	12	1 (8%)	11	< 0.01
Gefitinib as 2nd time use	3	3 (100%)	0	
Erlotinib as first time use	12	1 (8%)	11	
Erlotinib as 2nd time use	3	2 (67%)	1	
Afatinib as first time use	12	2 (17%)	10	
Dacomitinib as first time use	1	0 (0%)	1	
Osimertinib as first time use	4	3 (75%)	1	
Osimertinib as 2nd time use	11	1 (9%)	10	
Therapeutic line, 2/3/4/5/6/7th	17/18/12/7/0/4	5/5/2/1/0/0	12/13/10/6/0/4	0.66
Treatment with corticosteroid, dosage of prednisolone at the start of TKI re-administration (mg)				
Yes / no	17 / 41	4 / 9	13 / 32	1.00
0/2.5/5/10/15/20/over 25	41/3/3/3/3/2/3	9/0/2/0/1/1/0	32/3/1/3/2/1/3	
Response				
PR/SD/PD/NE	28/20/3/7	5/5/1/2	23/15/2/5	0.74
Response rate (%)/Disease control rate (%)	55/94	45/91	58/95	0.51
Status of initial TKI-induced ILD				
CTCAE Grade, 1/2/3/4	26/20/11/1	5/6/2/0	21/14/9/1	0.75
Onset, days from initial TKI, median (range)	61 (4–537)	51 (15–537)	61 (4–457)	0.42
Radiological pattern of initial TKI-induced ILD, DAD/non-DAD/undetermined	4/53/1	0/13/0	4/40/1	0.56
Dosage of corticosteroid (prednisolone, mg), median (range)	45 (10–70)	40 (20–60)	50 (10–70)	0.45
Duration of corticosteroid, median (range), days, median (range)	56 (7–511)	52 (24–112)	70 (7–511)	0.07
Duration between stopping of corticosteroid and TKI re-administration, days, mean (range)	96 (0–853)	66 (0–461)	99 (0–853)	0.67
Duration between onset of initial TKI-induced ILD and TKI re-administration, days, median	119 (7–892)	76 (20–594)	128 (7–892)	0.70
Radiological finding at TKI re-administration, remained/disappeared	27/31	8/5	19/26	0.22

*CTCAE* common terminology criteria for adverse events, *CR* complete response, *DAD* diffuse alveolar damage, *ECOG* Eastern Cooperative Oncology Group, *ILD* interstitial lung disease, *NE* not evaluated, *PD* progressive disease, *PR* partial response, *SD* stable disease, *TKI* tyrosine kinase inhibitor

*At diagnosis for lung cancer

^a^One patient was treated with addition of bevacizumab

^b^Two patients were treated with addition of ramucirumab

**Fig. 1:**
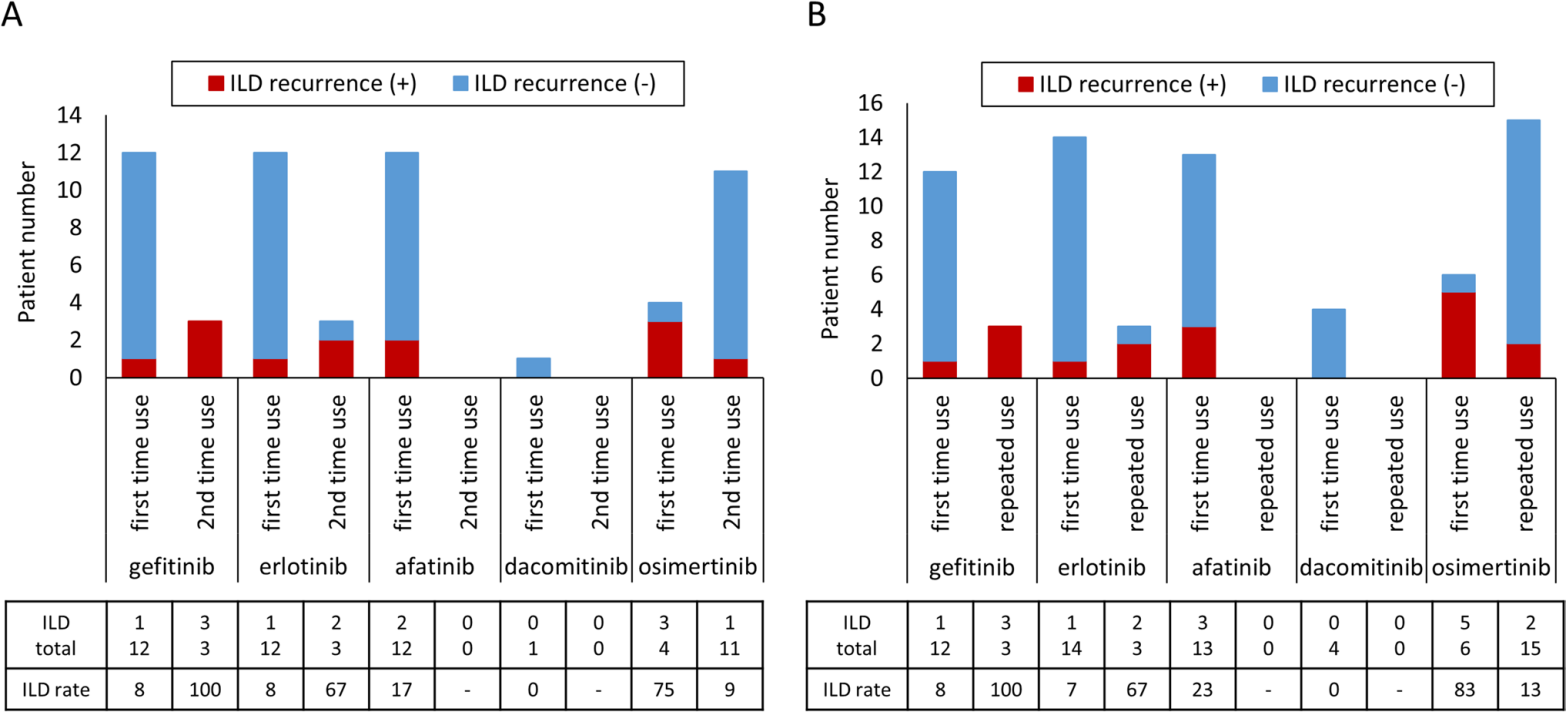
Types of EGFR-TKI used and incidences of ILD. **A** After EGFR-TKI re-administration. **B** After third TKI use (including re-administration). First time use: when a TKI was used for the first time at that time. Second time use: when the same TKI was used as the initial one. Repeated use: when a previously used TKI was used again

We examined factors involved in the development of TKI re-administration-induced ILD (Table 3). There were no significant differences in patient characteristics and status of TKI re-administration such as smoking history, performance status, therapeutic line, concomitant corticosteroid use, T790M, and status of initial TKI-induced ILD. Thirty cases received cytotoxic chemotherapy between initial TKI administration and re-administration: platinum-doublets were the first choice in all cases, followed by other cytotoxic agents in 13 cases. There was no difference in such therapeutic situation between TKI re-administration-induced ILD and no ILD groups. There was no difference in the incidence of ILD by type of TKI at re-administration. However, the incidence of ILD was different depending on the administration pattern of the initial and second TKIs (Table 3 and Fig. 1A). At re-administration, gefitinib was selected in 12 patients as the first time use; that is, these 12 patients were initially treated with a TKI other than gefitinib. ILD was observed in one patient (ILD recurrence rate was 8%). In three patients, the initial TKI was gefitinib and the second TKI was also gefitinib (second time use); ILD was observed in all three patients (ILD recurrence rate was 100%). Similar to gefitinib, ILD recurrence was observed in 1 (8%) of 12 patients who were initially treated with erlotinib and in 2 (67%) of 3 in patients re-treated with erlotinib (second time use). In contrast, ILD was observed in 3 (75%) of 4 in patients treated with osimertinib as the first time use and in 1 (9%) of 11 in patients treated with osimertinib as second time use. This administration pattern of TKIs was associated with ILD recurrence (*p* < 0.01).

### Efficacy of EGFR-TKI Re-administration

**Fig. 2:**
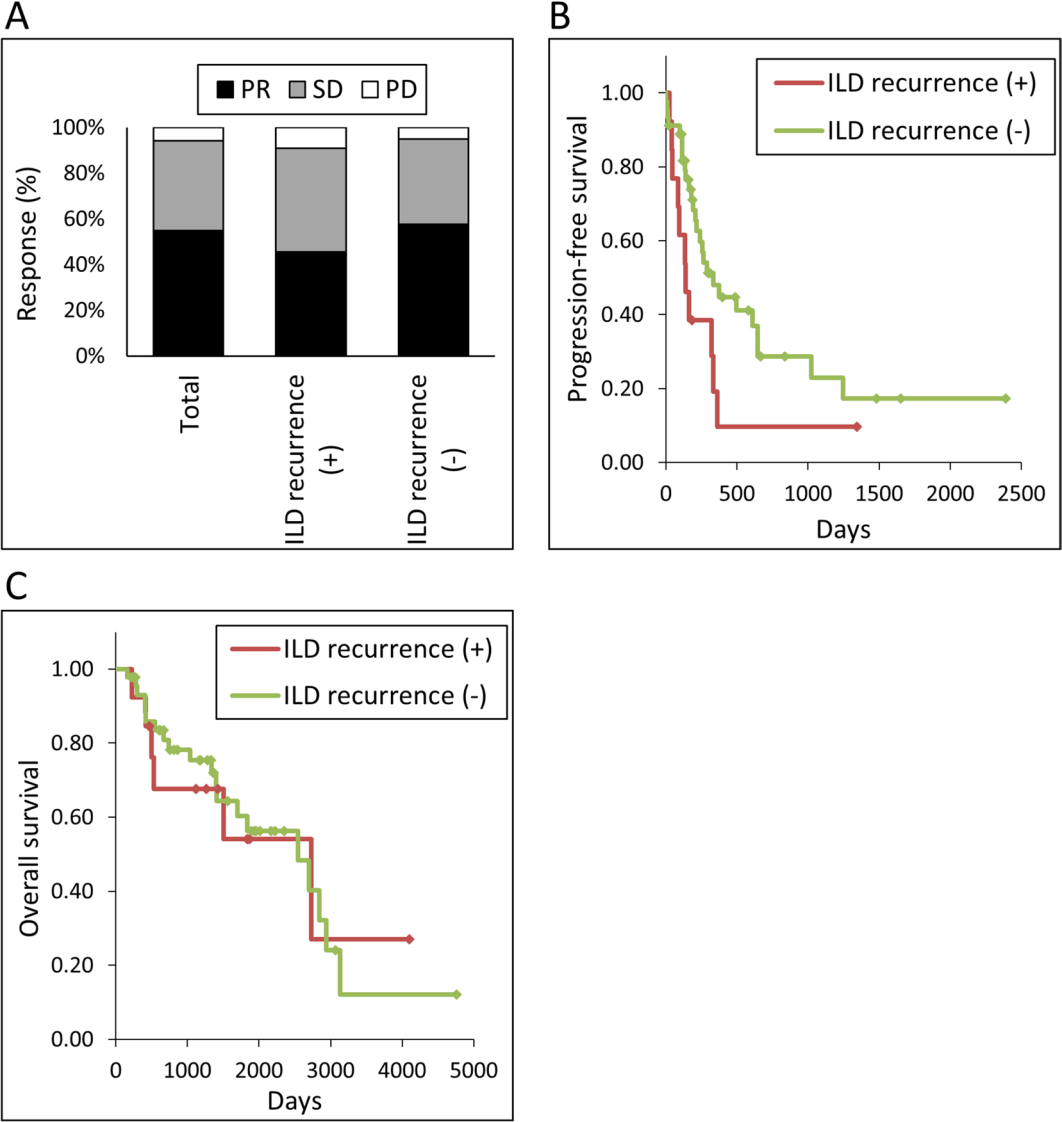
Efficacy of EGFR-TKI re-administration. **A** Response. PR: partial response. SD: stable disease. PD: progressive disease. **B** Kaplan–Meier curves of progression-free survival. **C** Kaplan–Meier curves of overall survival

The response rate to EGFR-TKI re-administration in the total population was 55, 45% in patients with ILD recurrence, and 58% in patients without ILD recurrence (Table 3 and Fig. 2A). Disease control rates were 94, 91, and 95% in total patients, patients with ILD, and patients without ILD, respectively. Kaplan–Meier curves for PFS of TKI re-administration are shown in Fig. 2B. The median PFS was 9.6 months in the total patient group. PFS tended to be longer in patients without ILD recurrence (median 11.1 months) than in patients with ILD recurrence (median 4.6 months), but this difference was not statistically significant (*p* = 0.05). The median OS was 84.8, 91.1, and 84.8 months in the total patient group and patients with and without ILD recurrence, respectively (Fig. 2C).

### Third EGFR-TKI Treatment (Second EGFR-TKI Re-administration)

Twelve patients received a third TKI treatment (Table 4); of these, two patients experienced TKI re-administration-induced ILD. Third TKI-induced ILD was observed in four patients (33%). Of six patients treated with osimertinib, ILD was observed in three patients (50%). ILD occurred in both patients who were treated with osimertinib for the first time. Thus, similar to the TKI re-administration, a higher incidence of ILD was also observed in third TKI administration when osimertinib was used for the first time. Third TKI-induced ILD grades were grade 2 in two patients and grade 3 in two patients. There were no other serious adverse events in response to the third TKI. The response rate was 22% (two of nine patients with available data) and DCR was 89% (eight of nine patients). Figure 1B shows TKI administration patterns and ILD recurrence rates by the 70 administrations of TKI, including both re-administration and third-time use. In patients with a history of another TKI-induced ILD treated with osimertinib for the first time, ILD recurred in 5 (83%) of six patients. The ILD recurrence rate was less than 10% when patients switched to gefitinib or erlotinib from other TKIs.

**Table 4:** Patients who received third EGFR-TKI

Case	Initial TKI	Re-administered TKI		Third TKI										
		TKI	ILD	TKI	Therapeutic line	Concurrent prednisolone (mg)	PS	ILD	ILD onset, days from TKI start	ILD grade	ILD radiological pattern	Corticosteroid for ILD	Response	PFS (days)
1	Osi	Osi	No	Dac	6	5	1	No					NE	12
2	Osi	Gef	No	Dac	4	0	1	No					PD	112
3	Erl	Gef	Yes	Afa	5	0	2	Yes	62	3	AEP	No	NE	99
4	Osi	Afa	No	Osi	4	0	1	No					SD	125
5	Erl	Gef	No	Osi	3	0	0	Yes	201	2	AEP	Yes	PR	319
6	Osi	Dac	No	Erl*	6	0	1	No					SD	274
7	Osi	Afa	No	Dac	9	10	1	No					NE	134
8	Osi	Erl	No	Osi	3	0	1	Yes	80	3	DAD	Yes	SD	185
9	Osi	Afa	No	Osi	5	0	0	No					SD	215^a^
10	Osi	Afa	No	Erl	4	0	1	No					SD	298
11	Afa	Erl	No	Osi	5	0	1	Yes	285	2	HP	Yes	PR	421^a^
12	Afa	Osi	Yes	Osi	5	0	1	No					SD	84

*AEP* acute eosinophilic pneumonia, *Afa* afatinib, *Dac* dacomitinib, *DAD* diffuse alveolar damage, *Erl* erlotinib, Gef: gefitinib, *HP* hypersensitivity pneumonia, *ILD* interstitial lung disease, *NE* not evaluated, *Osi* osimertinib, *PD* progressive disease, *PFS* progression-free survival, *PR* partial response, *SD* stable disease, *TKI* tyrosine kinase inhibitor

*With ramucirumab

^a^Non-PD at the evaluated day

## Discussion

In the present study, we investigated 58 patients who were re-treated with EGFR-TKI after recovery from EGFR-TKI-induced ILD; this is the largest study to date to address this question. We found that (1) the ILD recurrence rate induced by EGFR-TKI re-administration was 22.4%; (2) ILD recurrence was frequently observed when gefitinib or erlotinib was used for the second time and when osimertinib was used for the first time; (3) conversely, ILD recurrence rate was low when gefitinib or erlotinib was used for the first time and when osimertinib was used for the second time; (4) EGFR-TKI re-administration-induced ILDs were grade 1–3, which was tolerable; and (5) the overall response rate, disease control rate, median PFS by TKI re-administration, and median OS were 55%, 94%, 9.6 and 84.8 months, respectively. Based on reasons above mentioned (1), (4), and (5), EGFR-TKI re-administration is feasible.

A multicenter retrospective cohort study analyzed 33 cases of osimertinib continuation (*n* = 21) or re-administration (*n* = 12) after osimertinib-induced pneumonitis [13]. Five patients (15%) re-experienced pneumonitis (grade 1 in three patients and grade 2 in two patients). The median PFS after osimertinib re-administration was not achieved (95% confidence interval: 10.3 months—not reached). The authors concluded that osimertinib re-administration was feasible and effective, and osimertinib may be considered a treatment option even after the development of mild pneumonitis [13]. Consistent with this result, in the current study, in patients treated with osimertinib after osimertinib-induced ILD, the frequency of ILD recurrence was low. The current study provides new evidence on EGFR-TKI re-administration in this population. Administration of gefitinib or erlotinib after osimertinib-induced ILD resulted in the lowest rate of ILD recurrence. This trend remained unchanged after the third use of TKI. The frequency of ILD recurrence was lowest when gefitinib (8%) or erlotinib (7%) was used for the first time, followed by repeated use of osimertinib (13%). First time use of osimertinib was associated with the highest frequency of ILD recurrence (five of six cases, 83%). These results are helpful in determining which TKI to re-administer with after osimertinib-induced ILD because osimertinib is currently often selected as the initial TKI therapy. Additionally, there was no difference in ILD recurrence rates by treatment line of TKI re-administration (5/17 (29%) in second-line treatment and 8/41 (20%) in third-line or thereafter). There is no evidence that platinum combination chemotherapy should be given before TKI re-administration. In turn, there is no evidence that platinum combination chemotherapy but not TKI re-administration is the standard therapy after TKI-induced ILD. Conversely, however, there is also no evidence that TKI re-administration should be preferred before chemotherapy. No conclusions can be drawn on this issue from this study. It must be important to use up both EGFR-TKI and platinum combination chemotherapy.

Classically, two mechanisms involved in drug-induced ILD have been proposed: one is direct, dose-dependent toxicity, and the other is immune-mediated toxicity [14]. Various additional factors may be associated with the onset of EGFR-TKI-induced ILD. EGFR-TKIs block EGFR phosphorylation and prevent the regeneration and proliferation of the injured epithelium [15]. The interruption of damage-repair mechanisms by EGFR activation may result in ILD [15]. Inflammation is also involved in ILD development. It is reported that transforming growth factor-β and its downstream interleukin-6 are involved in EGFR-TKI-induced ILD [16].

Previous case series reports have suggested that EGFR-TKI re-administration with corticosteroids can prevent the recurrence of ILD because of the anti-inflammatory action of corticosteroids [17, 18]. In the current study, all consecutive eligible patients within the study period were recruited. Corticosteroids were used concomitantly in 17 of 58 patients, and there was no difference in ILD recurrence rates with or without corticosteroids. Although the doses and duration of corticosteroids varied, this result did not indicate a benefit of concomitant steroid use for preventing ILD recurrence. However, we cannot conclude that corticosteroid is unnecessary for the prevention of ILD. There are multiple mechanisms of ILD, and the proportion of each mechanism may vary from case to case. Corticosteroids may be effective in preventing the development of ILD in some cases.

The anti-tumor effect of TKI re-administration was significant. The response rate was 55%, DCR was 94%, and median PFS was 9.6 months. These effects tended to be better in the absence of ILD recurrence. Importantly, even when ILD recurrence was observed, these effects were not significantly worse. The median OS was 91.1 and 84.8 months in patients with and without ILD recurrence, respectively. The presence of driver gene mutations, including the *EGFR* gene, is a favorable prognostic factor in NSCLC [19], and the prognosis is shorter when *EGFR* mutations are present but EGFR-TKI is not used [20]. While there is no information for outcomes in patients who did not receive TKI re-administration, TKI re-administration likely contributed to long-term OS in most patients assessed in this study.

This study has several limitations. First, EGFR-TKI-induced ILD was diagnosed on the basis of patients’ clinical courses and radiological findings by the respiratory specialist at each institute but not by central judgment. Therefore, the diagnosis and radiological type might be inaccurate in part. Conversely, the diagnosis and management of ILD in this study is the very essence of usual medical practice and is indeed useful information for future treatment policy. Second, this was conducted as a retrospective study. There is likely a selection bias of patients who received EGFR-TKI re-administration. Third, recurrence rate of ILD in first time osimertinib receivers was high, but it might be inaccurate because of small number of cases in this group. There is also a TKI selection bias based on the T790M status and other factors. Fourth, no information on the incidence of initial EGFR-TKI-induced ILD was available.

## Conclusions

This study showed that EGFR-TKI re-administration is a feasible and effective treatment for patients who experienced EGFR-TKI-induced ILD. Our results indicate that re-administration of EGFR-TKI is an important option for long-term prognosis after recovery from EGFR-TKI-induced ILD.

## Data Availability

There is no data that can be provided other than Figures and Tables.
